# The Canine Gut Health: The Impact of a New Feed Supplement on Microbiota Composition

**DOI:** 10.3390/ani14081189

**Published:** 2024-04-15

**Authors:** David Atuahene, Ibrahim Zuniga-Chaves, Elisa Martello, Bruno Stefanon, Garret Suen, Fatemeh Balouei, Giorgia Meineri

**Affiliations:** 1 Department of Veterinary Sciences, School of Agriculture and Veterinary Medicine, University of Turin, 10095 Grugliasco, Italy; giorgia.meineri@unito.it; 2 Department of Bacteriology, University of Wisconsin-Madison, Madison, WI 53706, USA; zunigachaves@wisc.edu (I.Z.-C.); gsuen@wisc.edu (G.S.); 3 Microbiology Doctoral Training Program, University of Wisconsin-Madison, Madison, WI 53706, USA; 4 Division of Epidemiology and Public Health, School of Medicine, University of Nottingham, Nottingham NG7 2UH, UK; martello.elisa@gmail.com; 5 Department of Agrifood, Environmental and Animal Science, University of Udine, 33100 Udine, Italy; bruno.stefanon@uniud.it (B.S.); balouei.fatemeh@spes.uniud.it (F.B.)

**Keywords:** gut microbiota, dogs, bromelain, quercetin, *Lentinula edodes*, healthy

## Abstract

**Simple Summary:**

The community of microorganisms and their genes in the gastrointestinal (GI) tract, collectively known as the gut microbiome, play multiple roles in canine health. Disruption, due to the lack of beneficial organisms or the overgrowth of pathogenic organisms, of the delicate balance of the microbes in the canine GI microbiome can lead to dire consequences including inflammatory bowel disease and diarrhea. Here, we provide evidence that a novel dietary supplement rich in antioxidants can stimulate the proliferation of beneficial bacteria, such as those in the genera *Bifidobacterium* and *Lactobacillus*. These bacteria are considered important for their anti-inflammatory activity and do not disrupt the overall gut microbiome composition of healthy adult dogs when compared to a controlled group over a period of time.

**Abstract:**

This study aimed to determine the impact of a novel formulation of a supplement composed of the natural ingredients, bromelain, quercetin, and *Lentinula edodes*, on the gut microbiota of healthy adult dogs. Adult healthy female dogs were administered either a placebo (CTR, *n* = 15) or the supplement (TRT, *n* = 15) over 28 days. Stool samples were collected for 16S rRNA sequencing before supplement administration (T0), at completion of supplement administration (T28), and one week after the end of supplement administration (T35) to characterize changes in the gut microbial communities. QIIME was used to determine both alpha- and beta-diversity, and ANCOM-BC was used to identify differences in taxonomic abundances before and after supplementation. We found a significant decrease in overall diversity in the CTR group but no significant differences in overall diversity in the TRT group over time. Furthermore, we found differences in the abundance of several taxa in both the CTR and TRT groups, but differences in the abundance of beneficial bacteria were more pronounced in the TRT group. Specifically, we found increases in the abundance of sequences belonging to the genera *Bifidobacterium*, *Lactobacillus*, and *Pediococcus* at T28 in the TRT group with significant increases in *Bifidobacterium* and *Lactobacillus* persisting at T35 when compared to T0. Importantly, members of these genera are considered important for their anti-inflammatory properties, vital for fostering a balanced and robust gut microbiota in dogs. The results of our study show the potential of our supplement to selectively enhance specific beneficial bacterial taxa, offering a targeted approach to modulating the gut microbiome without causing disruptions to the overall equilibrium.

## 1. Introduction

Research on gut microbiota has attained attention in recent years largely because of its relationship to gastrointestinal (GI) and extra gastrointestinal (extra-GI) diseases. The gut microbiota refers to the group of microorganisms such as bacteria, viruses, archaea, protozoa, and fungi that coexist symbiotically in the host GI tract [1] They perform various functions in several important processes including energy metabolism, neuronal activity, immunomodulation, digestion, fermentation, and protection against pathogens. Non-digestible compounds in food, such as plant-derived polysaccharides, and nutrients that escape from small intestinal digestion can also be degraded by these microorganisms. Moreover, the gut microbiota also plays an important role in the homeostasis of the host by modulating the function of several vital systems, such as the cardiovascular, renal, and immune systems [1,2,3].

From an immunological perspective, specific microorganisms can be recognized as pathogenic by the host immune system. However, the majority of gut microorganisms are non-pathogenic and, instead, co-exist with enterocytes in an interdependent relationship [4]. A balanced and well-functioning microbiota can be both pro-inflammatory and anti-inflammatory to maintain a balance that avoids over-inflammation while responding quickly to infections [5]. The types of microorganisms that make up the microbiota vary from site to site and species to species [6,7].

In animals, the health status of the gut microbiome is connected to a variety of diseases. For example, dysbiosis, or an imbalance in the microbiome, has been associated with a number of inflammatory diseases such as inflammatory bowel disease (IBD) and leaky gut syndrome in dogs. Previous research reported that dogs living in breeding conditions are more vulnerable than their companions to chronic stress, which is linked to more gut disorder occurrences and increased dysbiosis. Intestinal dysbiosis is frequently associated with a decrease in microbial diversity in dogs with IBD [8]. Diarrhea, colitis, and constipation are other clinical symptoms prevalent in millions of humans and dogs around the world, and changes in the gut microbiota play a significant role in the onset and progression of these conditions [1,2]. Similarly, an imbalanced gut microbiota in dogs has been linked to metabolic complications within the gut such as impaired nutrition absorption, altered metabolism of dietary components, and many other GI disorders in which a classical treatment would involve the use of antimicrobial drugs and antibiotics [2,3]. However, it should be noted that antibiotic treatment increases the risk of developing antimicrobial resistance (AMR) and poses threats on gut microbiota such as reduction in species diversity, alteration of metabolic activity, and consequent antibiotic-resistant organisms, which in turn can lead to antibiotic-associated diarrhea and recurrent infections [3,9]. Thus, there is a growing need for treatments that do not rely on antimicrobials, such as the use of natural agents, which do not pose a risk of AMR [10,11].

Nutraceuticals, encompassing a wide range of natural substances with health benefits and fewer side effects, offer a promising alternative. They provide health benefits and aid in the prevention of diseases [12]. A large variety of nutraceutical groups have antioxidant activity, making them useful agents in a variety of disorders [13] which include oxidative stress, a major component in several gastrointestinal disorders [14]. Among these nutraceuticals are bromelain (B), *Lentinula edodes* (LE), and quercetin (Q).

Bromelain (B) is a complex mixture of enzymes and bioactive compounds naturally occurring in various parts of pineapples, including their stems, fruits, and roots [15]. Among its constituents, proteinases and proteases are the key enzymes responsible for protein breakdown within the body. Notably, bromelain can easily be absorbed from the gut without interfering with protein digestion in the gastrointestinal tract. In addition to its antibacterial and anti-inflammatory properties, bromelain has indicated efficacy in managing various gastrointestinal ailments such as dyspepsia [16].

Quercetin (Q) is a significant polyphenolic flavonoid, present in a wide range of fruits and vegetables, including apples, onions, grapes, berries, and capers [17]. Human studies have indicated that quercetin, in addition to being a potent antioxidant, has valuable anti-inflammatory qualities [13]. Additionally, it has been shown to improve intestinal dysbiosis (HFD) in mice fed with a high-fat diet by attenuating lipoprotein-dependent activation of the lipoprotein-dependent pathway (TLR-4) [18].

Lastly, mushrooms contain a wide variety of bioactive substances including polysaccharides, dietary fibers, minerals, and proteins [19,20]. *Lentinula edodes* (LE), commonly known as the shiitake mushroom, is utilized in supplement preparations and has demonstrated anti-inflammatory, antioxidant, and anti-diabetic properties in studies involving both humans and animal models [21,22,23]. In addition to their biological effects, there has also been work focusing on regulating gut microbiota through the use of mushroom polysaccharides [24,25].

Here, we postulate that the formulated antioxidant-rich supplements positively influence the gut microbiota composition in healthy dogs. Here, we seek to evaluate the activity of a formulated antioxidant-rich supplement composed of the natural ingredients B, Q, and LE on the gut microbiota of healthy adult dogs and determine if this new formulation impacts microbiota stability in the gut when compared to a placebo.

## 2. Materials and Methods

### 2.1. Animals and Study Design

In a double-blinded randomized controlled trial, a total of 30 adult female American Staffordshire Terrier dogs, weighing 17 ± 1.5 kg and 5 ± 1 years old were selected from Ente Nazionale Cinofilia Italiana (ENCI) www.enci.it (accessed on 5 May 2022), a registered dog breeder in Italy. Animals participating in this study were the same as those reported in [14]. The study was performed in strict compliance with the guidelines of the Ministry of Health for the use of animals in research. Before obtaining formal informed consent, the overall objectives of the study were disclosed to the breeder. Both study protocols and the use of supplements were approved by the Ethical Animal Care and Use Committee of the University of Turin on 19 July 2022 under protocol number 2741.

The health status of all the animals was evaluated by a veterinarian through a general physical examination followed by a copromicroscopic analysis of their feces. All animals were found to be healthy with no underlying conditions that required treatment. The 30 dogs were randomly allocated to two groups: a control (CTR, *n* = 15) and treatment (TRT, *n* = 15) group. Dogs were housed in cages (3 dogs per cage), with an area of 6 ± 2 square meters. To ensure animal welfare following the principles of animal welfare legislation (Regolamento regionale 11 novembre 1993, Turin, Italy), dogs were provided with an open space of the same size as their box, thus avoiding public stress due to collective manipulation. Animals in both groups received a commercially available diet (“Royal Canin, Medium Adult Dry Dog Food”) (Supplementary Table S1) from 7 days prior to the commencement of the study to allow the GI system to adapt to the new diet. The daily calorie intake of each group was calculated as per FEDIAF Nutritional Guidelines (FEDIAF, 2021) using the following equation such that “ME” is metabolizable energy and “BW” is body weight:

ME (kcal/day) = 110 kg BW^0.75^

In both the CTR and TRT groups, each participant was given either a placebo of maltodextrin powder or a formulated feed containing a mixture of nutraceutical ingredients in their food once a day for 28 days. The dosage administered was 1 g/10 kg of BW [14].

### 2.2. Formulated Feed Composition

The formulated feed consisted of a mixture of nutraceuticals including 1.35% of bromelain (B) and quercetin (Q) each, and 1.00% *Lentinula edodes* mushrooms (LE). The exact compositions of the formulated feed were previously reported [14] and can be found in Supplementary Table S2.

### 2.3. Safety and Nutritional Parameters

An experienced veterinarian measured their body weight (BW) at T0 (baseline) and then at T28 (28 days after the beginning of the feed supplement administration) for all animals, consistently at the same time of day. A Body Condition Score (BCS), using a scale of 1 to 9, was determined through a physical (visual) examination and palpation at T0 and T28 by the same experienced veterinarian. Additionally, the fecal pH was determined using a calibrated pH instrument at T0 and T28 (HI 9125 pH/ORP meter; Hanna Instruments, Bedfordshire, UK). The data for these parameters have been reported previously [14].

### 2.4. Fecal Sample Collection

Fresh fecal samples were collected in a sterile plastic bag using a sterile spatula and temporarily held in a 4 °C freezer before moving into a −80 °C freezer until sequencing, as previously described [14]. To minimize bias, a non-biased blinded procedure was employed for the identification of all samples. Microbiome analysis was subsequently conducted on fecal samples collected at T0, T28, and T35.

### 2.5. Fecal DNA Extraction, PCR Amplification, and Amplicon Target Sequencing

Microbial DNA was extracted from a total of 150 mg of fecal samples using a DNA Miniprep kit (Zymo Research; Irvine, CA, USA) following the manufacturer’s protocol, including a bead beating step. The total DNA was quantified using a QubitTM 3 Fluorometer (Thermo Scientific; Waltham, MA, USA) and subjected to a PCR step, where the V3-V4 regions of the 16S rRNA gene were amplified using indexed primers [26] and DNA was purified using a Nextera DNA Library Prep Kit (Illumina; San Diego, CA, USA), according to the manufacturer’s instructions. The generated amplicons were sequenced on an Illumina MiSeq (San Diego, CA, USA) using a 2 × 250 bp paired-end v2 sequencing kit.

### 2.6. Computation and Sequence Analysis

The resulting raw sequences (FASTQ) were processed using the same standard operating procedure as previously described [27]. Briefly, sequences were filtered and processed using DADA2 in the Quantitative Insights Into Microbial Ecology 2 (QIIME 2) program [28]. Chimeric sequences were identified and subsequently removed from the dataset, and the remaining sequences were then grouped into Amplicon Sequence Variants (ASVs) and assigned taxonomy using the Greengenes database (version 13.8). The ASV abundance tables, the taxonomy tables, and the associated phylogenetic trees were imported into R v4.3.2 [29] and used to build a phyloseq object [30].

### 2.7. Taxonomic Statistical Analysis

The phyloseq object, containing the ASV abundances, was used to calculate alpha- and beta-diversity along with differential abundance analyses of taxa. For alpha-diversity, samples were rarefied down to the read depth of the sample with the lowest read depth. Shannon’s diversity, the observed species, and the inverse Simpson were the metrics calculated. Beta-diversity was conducted using principal coordinate analysis (PCoA) using the Bray–Curtis dissimilarity matrix in the vegan package [31]. The Bray–Curtis dissimilarity matrix was further used in a PERMANOVA analysis with the adonis function of the vegan package [32] with 1000 permutations. For diversity metrics, the threshold for significance was set at *p* < 0.05.

To examine if individual ASVs changed over time, differential abundance analysis was conducted using the ancombc2 function in the ANCOM-BC package [33] in R. *p*-values were adjusted using the false discovery rate procedure and ASVs with a corrected *p*-values < 0.1 were considered significant.

## 3. Results

A total of 5,346,677 reads were generated across the 90 samples. After filtering and sequence clean-up, an average read count per sample of 60,075 ± 4122 was retained, which clustered into 3038 distinct amplicon sequence variants (ASVs) that classified into 15 phyla, 103 families, and 153 genera. To ensure comprehensive alpha- and beta-diversity analyses, samples were rarefied based on the sample with the lowest read count, which was 51,499 reads. The rarefaction curves, depicted in Supplementary Figure S1, indicated that this read depth was sufficient to capture most of the diversity within the samples.

**Figure 1:**
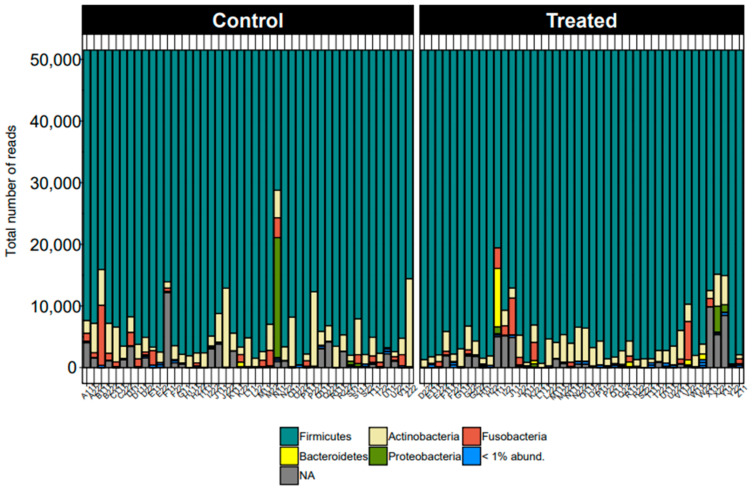
Taxonomic distribution for all dogs across samples from control and treatment groups by phyla.

At the phylum level, the taxonomic distribution displayed remarkable consistency across all samples. Firmicutes emerged as the predominant phylum, followed by Actinobacteriota and Fusobacteriota, as illustrated in Figure 1. This uniformity underscores the substantive presence and importance of these phyla within the studied microbiome.

**Figure 2:**
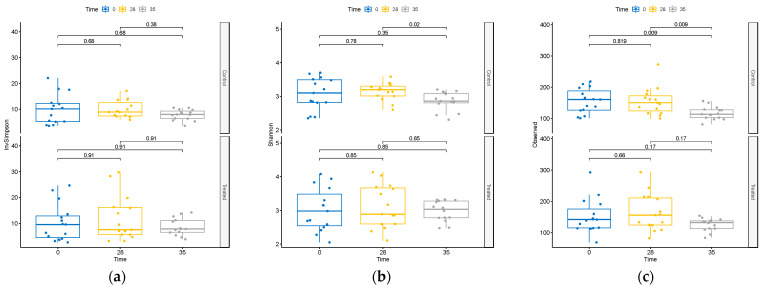
Changes in alpha-diversity as measured by (**a**) inverse Simpson index, (**b**) Shannon diversity index, and (**c**) observed ASVs at T0, T28, and T35 in treated and control groups.

The impact of the supplement on microbiome diversity was rigorously assessed. Comparative analysis across treatments at T0, T28, and T35 revealed no discernible differences in alpha-diversity metrics within the treatment group (Supplementary Figure S2). Moreover, as shown in Figure 2, within the TRT group, the assessment of diversity alterations over time revealed no significant changes in alpha-diversity.

Contrarily, within the CTR group, we observed a decline in alpha-diversity at day 35 (Figure 2). This decline was evident in both the total observed ASVs and the Shannon’s index, a widely used metric that accounts for species richness and evenness in data evaluation, highlighting a substantial reduction in diversity within this group at that specific time point.

**Figure 3:**
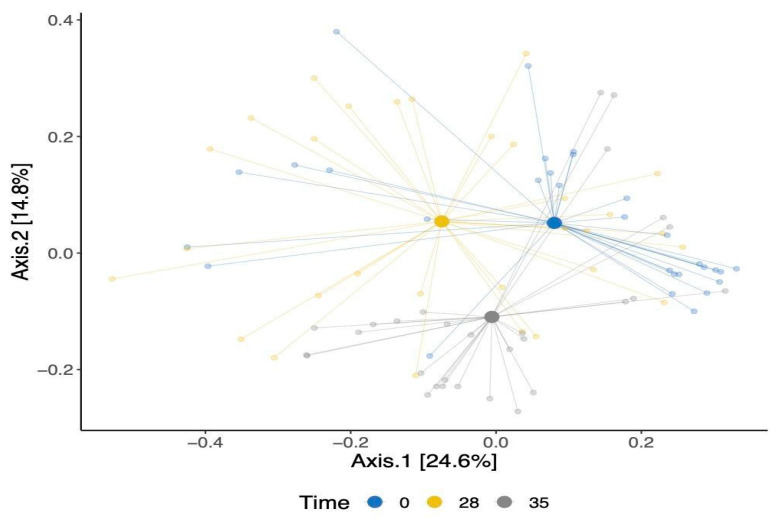
A principal coordinate analysis of Bray–Curtis’s dissimilarity matrix for β-diversity for all dogs colored by time points.

Our analysis of beta-diversity showed a shift in the overall gut bacterial communities over time (Figure 3). This was confirmed using an Adonis test, which showed that the observed separation is statistically associated with time point differences. The identified changes were evident in both the TRT and CTR groups, signifying shifts in the bacterial communities within these cohorts. Moreover, a modest statistically significant dissimilarity was noted when comparing the bacterial compositions of the dogs between the TRT and CTR groups (*p*-value = 0.02), as shown in Supplementary Figure S3.

We then identified ASVs that were determined to be differentially abundant in the TRT group between T0 and T28. Several ASVs were found to be differentially abundant in this group over time (Table 1), including increased abundance of ASVs belonging to *Bifidobacterium* (β = 0.065, SD = 0.014, corrected *p*-value < 0.001), *Lactobacillaceae* (unclassified at genus level) (β = 0.104, SD = 0.022, corrected *p*-value < 0.001), and *Peptostreptococcaceae* (unclassified at genus level) (β = 0.05, SD = 0.015, corrected *p*-value = 0.019). There was also a decrease in ASVs belonging to *Fusobacteria* (unclassified at species level) (β = −0.070, SD = 0.023, corrected *p*-value = 0.049), *Peptococcus* (β = −0.059, SD = 0.010, corrected *p*-value = 0.007), and *Dorea* (β = −0.053, SD = 0.015, corrected *p*-value = 0.019).

We also observed ASVs that were differentially abundant in the placebo group between T0 and T28 (Supplementary Table S2). These included increased abundances of ASVs belonging to *Bifidobacterium* (β = 0.019, SD = 0.007, corrected *p*-value = 0.081) and *Lactobacillaceae* (unclassified at genus level) (β = 0.084, SD = 0.027, corrected *p*-value = 0.058). There were also decreases in the abundances of ASVs belonging to *Fusobacteria* (unclassified at species level) β = −0.058, SD = 0.021, corrected *p*-value = 0.081). Although there were changes in both the TRT and CTR groups, differences in the CTR group were less pronounced than changes in the TRT group as observed by beta coefficients.

**Figure 4:**
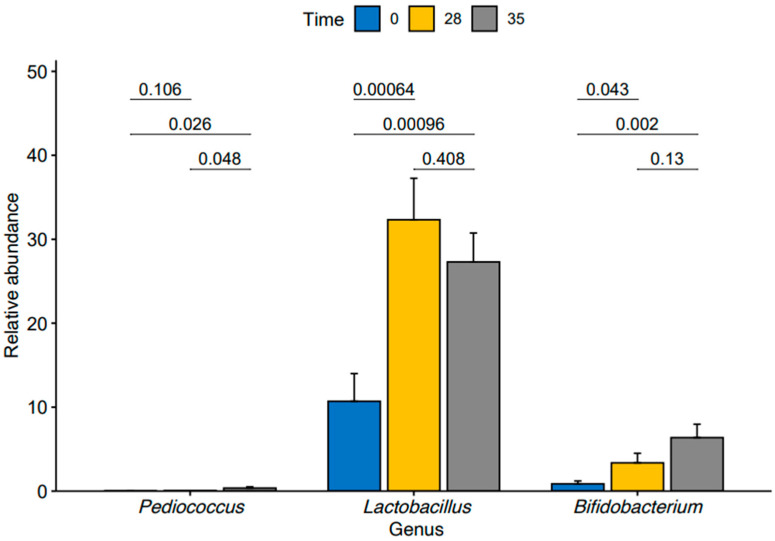
Relative abundance of *Pediococcus*, *Lactobacillus,* and *Bifidobacterium* at time points T0, T28, and T35 in treated group.

We then conducted a pairwise analysis of differences in relative abundance of the three enriched genera within our TRT group between time points using a pairwise *t*-test (Figure 4). We found a significant increase in *Lactobacillus* (*p*-value = 0.00064) and *Bifidobacterium* (*p*-value = 0.043) from T0 to T28. We also observed significant increases in the abundances of the *Lactobacillus* (*p*-value = 0.00096) and *Bifidobacterium* (*p*-value = 0.002) from T0 to T35.

## 4. Discussion

This study provides evidence that the use of a novel supplement that includes a blend of nutraceuticals in the form of B, Q, and LE results in a shift in the gut microbiome of healthy dogs after four weeks of treatment. Although we did not observe changes in alpha-diversity after the supplementation in the TRT group as measured by inverse Simpson’s Index, Shannon’s Diversity Index, or observed species, we found shifts in individual taxa over time. It has previously been shown that supplementation with this blend of nutraceuticals improves levels of stress and inflammation and also increases the presence of short-chain fatty acids (SCFAs) in this same cohort of dogs [14]. We postulate that the shift in the gut microbiome has a hand in modulating the improvement in these biomarkers. While there were decreases in the alpha-diversity over time, specifically in observed ASVs and Shannon Diversity in the control group, these differences were likely due to the natural fluctuations of the gut microbiome.

ASVs belonging to the families *Bifidobacteriaceae* and *Lactobacillaceae* were increased after supplementation administration in the TRT group. Members of *Bifidobacteriaceae,* and *Lactobacillaceae* include commensal bacteria that produce several SCFAs including propionate, acetate, and formate as products of carbohydrate fermentation [34]. While *Bifidobacteria* tend to be less abundant in dogs when compared to humans, it provides metabolites that are anti-inflammatory in dogs. For example, propionate, which decreases the production of cytokines, including IL-6 and IL-8, that promote inflammation [35]. We also observed decreases in ASVs belonging to *Peptococcus* and *Dorea longicatena*, both of which are SCFA producers and known to be residents in the guts of healthy canines under a state of eubiosis [36]. We postulate that the reduction in these groups of bacteria may be a result of the increase in other SCFA producers, including *Lactobacillaceae.* We note that Panasevich et al. [37] showed that a cohort of healthy adult Beagles administered nonviable *Lactobacillus acidophilus* (a species belonging to *Lactobacillaceae*) plus prebiotics had a significant increase in the total SCFAs, a significant increase in the abundance of bacteria belonging to *Lactobacillaceae*, and a decrease in the abundance of *Dorea* when compared to dogs on a control diet [37].

Additionally, the ASVs belonging to *Dorea longicatena* may have a role in levels of cortisol, a hormone that responds to stress, in the dogs. Associations between cortisol and gut microbial abundance in dogs have been explored [2,38]. In a randomized study of 20 5-month-old beagles examining the safeness of softening dog food with water, dogs that consumed softened dog food had higher levels of cortisol and higher levels of pathogenic bacteria including *Enterococcus* [38]. In our prior study, we found that cortisol decreased in our cohort of dogs 28 days after supplement administration [14]. Moreover, work has previously shown that a 35-day *Saccharomyces boulardii* supplementation resulted in a decrease in cortisol levels and a decrease in ASVs belonging to the genus *Dorea* [2] as in the current study.

We also observed a decrease in ASVs belonging to *Bacteroides* in the TRT group. *Bacteroides* are among the gut microbes that can metabolize tryptophan into various metabolites including indole and the indole-related skatole [39,40]. Indole is involved in intra-bacterial communication and has been linked to the reduction in motility [41], cytotoxicity [42], and invasiveness [43] of pathogenic bacteria. Despite these benefits, excess indole can be detrimental to the host. In studies involving rodent models, germ-free rodents that were colonized with indole-producing *E. coli* had significantly higher emotional responses to mildly stressful events in a study involving mice [44] and increased anxiety-like behavior in rats [45] when compared to rodents that were colonized with non-indole-producing *E. coli*. This provides a possible link between the decreased *Bacteroides* and our previous observation of decreased cortisol and the decreased indole in this cohort of dogs [14].

Our work here shows that the dogs in the TRT group had an increase in abundance of ASVs belonging to the *Lactobacillaceae* family. Members of the *Lactobacillaceae* family have been inversely associated with calprotectin, a protein that is released by neutrophils which are white blood cells. The presence of calprotectin in feces is indicative of gastrointestinal inflammation [46]. In our prior study, calprotectin was significantly reduced over the course of supplementation in dogs [14]. Similarly, a double-blind study of 22 dogs, given either a lactic-acid-bacteria (LAB) supplement containing *Limosilactobacillus fermentum*, *Lacticaseibacillus rhamnosus*, and *Lactiplantibacillus plantarum* or a placebo over 7 days showed that all the dogs given the LAB supplement had negligible levels of calprotectin while five dogs given the placebo had increased calprotectin after supplementation [47].

Our study used a double-blinded randomized controlled design, a study design that reduces possible bias between observed differences in the treatment and placebo group. However, this study is limited by the use of 16S rRNA sequencing, which can reveal only the structural profiles of the gut microbial community. To examine functional aspects of the gut microbiome, whole-genome metagenomics, metatranscriptomics, or metaproteomics would need to be conducted. However, we note that we have previously reported on fecal measures [14], including the sum of all SCFA that are bacteria-derived. This information provides valuable insights into the functional roles of certain members of the gut microbiome. An additional limitation of this study is that we observed the changes in the features of the microbiome over time in the placebo group, namely, decreases in observed species after administration of the placebo supplement, in addition to changes in individual ASV abundances over the course of placebo supplementation. These differences are likely a result of natural fluctuations in the gut microbiome over time. Despite observing changes in the placebo group, the differences observed in ASV abundances were more pronounced in the TRT group, indicating that the supplement is likely a contributor to the observed shift in the microbiome. We also found that there is an overall difference in the microbiotas between the two groups (Supplementary Figure S3), suggesting a nuanced but noteworthy divergence between the microbiotas of these distinct experimental cohorts.

## 5. Conclusions

Our study demonstrates that a supplement containing Q, B, and LE alters the gut microbiota of adult dogs after a 4-week treatment. Although it promotes the growth of various microbes, the supplement does not disturb the overall gut microbiome diversity. This suggests its potential as a microbial modulator without compromising the overall balance, making it a candidate for investigating its role alongside or in support of antibiotics.

## Figures and Tables

**Table 1:** Differentially abundant ASVs by time within treated dogs as determined by ANCOM-BC (T0 vs. T28).

Taxonomic Classification	Higher Abundance Time Point	Beta Coefficient	Standard Deviation	W Statistic	*p*-Value	Corrected *p*-Value	ASV Code
*Fusobacteria*_unclassified	T0	−0.047	0.016	−2.972	0.003	0.050	ASV334
*Fusobacteria*_unclassified	T0	−0.070	0.023	−3.025	0.002	0.049	ASV029
*Bifidobacterium* sp.	T28	0.065	0.014	4.698	0.000	0.000	ASV073
*Bifidobacterium animalis*	T28	0.089	0.023	3.925	0.000	0.005	ASV117
*Bifidobacterium breve*	T28	0.070	0.023	2.993	0.003	0.050	ASV007
*Lactobacillaceae*_unclassified	T28	0.104	0.022	4.697	0.000	0.000	ASV256
*Lactobacillaceae*_unclassified	T28	0.063	0.017	3.827	0.000	0.006	ASV585
*Lactobacillaceae*_unclassified	T28	0.111	0.025	4.489	0.000	0.001	ASV098
*Lactobacillaceae hamsteri*	T28	0.098	0.027	3.568	0.000	0.012	ASV440
*Anaerorhabdus furcosa*	T28	0.055	0.016	3.318	0.001	0.022	ASV269
*Bacillaceae*_unclassified	T28	0.047	0.016	2.847	0.004	0.070	ASV706
*Lactobacillus hamsteri*	T28	0.064	0.023	2.715	0.007	0.099	ASV408
*Peptococcus* sp.	T0	−0.059	0.016	−3.590	0.000	0.012	ASV519
*Peptostreptococcaceae*_unclassified	T28	0.050	0.015	3.387	0.001	0.019	ASV003
*Peptostreptococcaceae*_unclassified	T28	0.046	0.015	3.140	0.002	0.037	ASV006
*Dorea longicatena*	T0	−0.053	0.015	−3.418	0.001	0.019	ASV262

## Data Availability

Data available upon request from the first author.

## Supplementary Materials

The following supporting information can be downloaded at: https://www.mdpi.com/article/10.3390/ani14081189/s1, Figure S1: Rarefaction curves for all samples for treated and control groups; Figure S2: Alpha-diversity analysis of treated vs control groups at T0, T28 and T35; Figure S3: A principal coordinate analysis using Bray–Curtis dissimilarity matrix for β-diversity colored by treated and control groups; Table S1: Guaranteed Analysis of commercially available diet provided to both groups; Table S2: Composition of nutraceutical and placebo. Table S3: Differentially abundant ASVs by time within dogs in the control group as determined by ANCOM-BC (T0 vs. T28).
