# Supplementary material for: The Canine Gut Health: The Impact of a New Feed Supplement on Microbiota Composition
Source: Animals (Basel). 2024 Apr 15;14(8):1189. doi: 10.3390/ani14081189 (PMC11047554; doi:10.3390/ani14081189)
Supplement: Supplementary file 1 [file animals-14-01189-s001.zip › animals-2926706-supplementary.pdf]

animals

Article

The Canine Gut Health: Impact of a New Feed Supplement on Microbiota Composition

Supplementary Materials

Supplemental Table S1: Guaranteed Analysis of commercially available diet provided to both groups.

New Formulation Ingredients (mg/g)	
Crude Protein (min.)	22.0%
Crude Fat (min.)	12.0%
Crude Fiber (max.)	4.1%
Moisture (max.)	10.0%
Eicosapentaenoic Acid (EPA) (min.)	0.17%
Docosahexaenoic Acid (DHA) (min.)	0.07%
Glucosamine* (min.)	818 mg/kg
Chondroitin sulfate* (min.)	8 mg/kg

***Not recognized as an essential nutrient by the AAFCO Dog Food Nutrient Profiles**

Supplemental Table S2: Composition of the tested supplement and the placebo.

New Formulation Ingredients (mg/g)	
Lentinula edodes	10.0
Quercetin	13.5
Bromelain	13.5
Maltodextrin	583.4
Appetite stimulants	379.6
Total	1000.0
**Placebo ingredients (mg/g)**	
Maltodextrin	1000.0

Supplemental Table S3: Differentially abundant ASVs by time within dogs in the control group as determined by ANCOM-BC (T0 vs T28).

Taxonomic classification	Higher Abundance Timepoint	Log2-Fold Change	Standard deviation	W statistic	p-value	Corrected p-value	ASV_code
Lachnospiraceae_unclassified	T0	-0.040	0.014	-2.862	0.004	0.081	ASV002
Clostridiales_unclassified	T0	-0.036	0.013	-2.767	0.006	0.081	ASV349
Lachnospiraceae_unclassified	T0	-0.054	0.013	-4.228	0.000	0.006	ASV781
Ruminococcus lactaris	T28	0.037	0.012	3.114	0.002	0.058	ASV969
Fusobacteria_unclassified	T0	-0.058	0.021	-2.752	0.006	0.081	ASV029
Collinsella stercoris	T28	0.042	0.015	2.808	0.005	0.081	ASV259
Paraeggerthella hongkongensis	T28	0.045	0.017	2.670	0.008	0.089	ASV527
Coriobacteriaceae_unclassified	T28	0.021	0.008	2.697	0.007	0.088	ASV345
Bifidobacterium sp	T28	0.019	0.007	2.786	0.005	0.081	ASV018
Bifidobacterium animalis	T28	0.054	0.020	2.743	0.006	0.081	ASV117
Bifidobacterium sp	T28	0.047	0.016	3.035	0.002	0.058	ASV772
Lactobacillaceae_unclassified	T28	0.084	0.027	3.132	0.002	0.058	ASV256
Lactobacillaceae_unclassified	T28	0.057	0.016	3.565	0.000	0.029	ASV585
Lactobacillaceae_unclassified	T28	0.100	0.029	3.418	0.001	0.038	ASV098
Lactobacillus hamsteri	T28	0.081	0.025	3.211	0.001	0.058	ASV440
Streptococcus equi	T28	0.080	0.026	3.042	0.002	0.058	ASV222
Anaerorhabdus furcosa	T0	-0.034	0.012	-2.818	0.005	0.081	ASV086
Eubacterium sp	T0	-0.048	0.018	-2.660	0.008	0.089	ASV825
Erysipelotrichaceae_unclassified	T28	0.056	0.021	2.644	0.008	0.089	ASV255
Enterococcus cecorum	T28	0.035	0.012	2.836	0.005	0.081	ASV036
Pediococcus sp	T28	0.050	0.016	3.081	0.002	0.058	ASV844
Lactobacillus sp	T28	0.102	0.026	3.880	0.000	0.013	ASV046

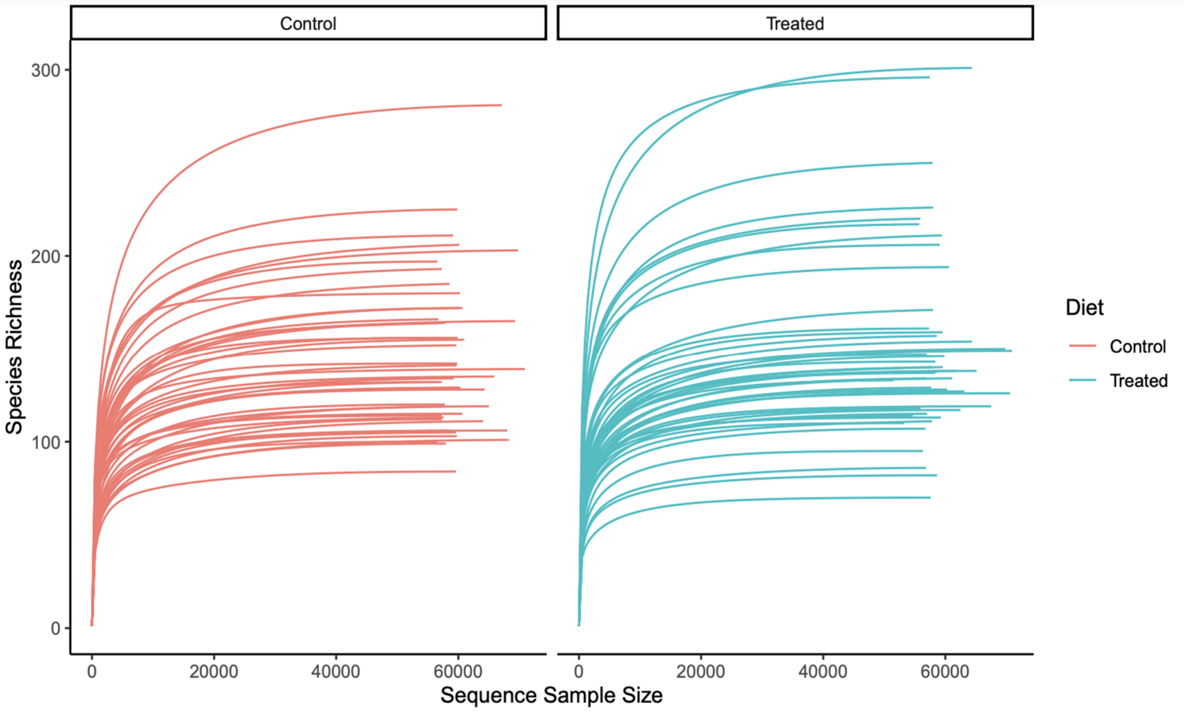

Supplementary Figure S1: Rarefaction curves for all samples for treated and control groups.

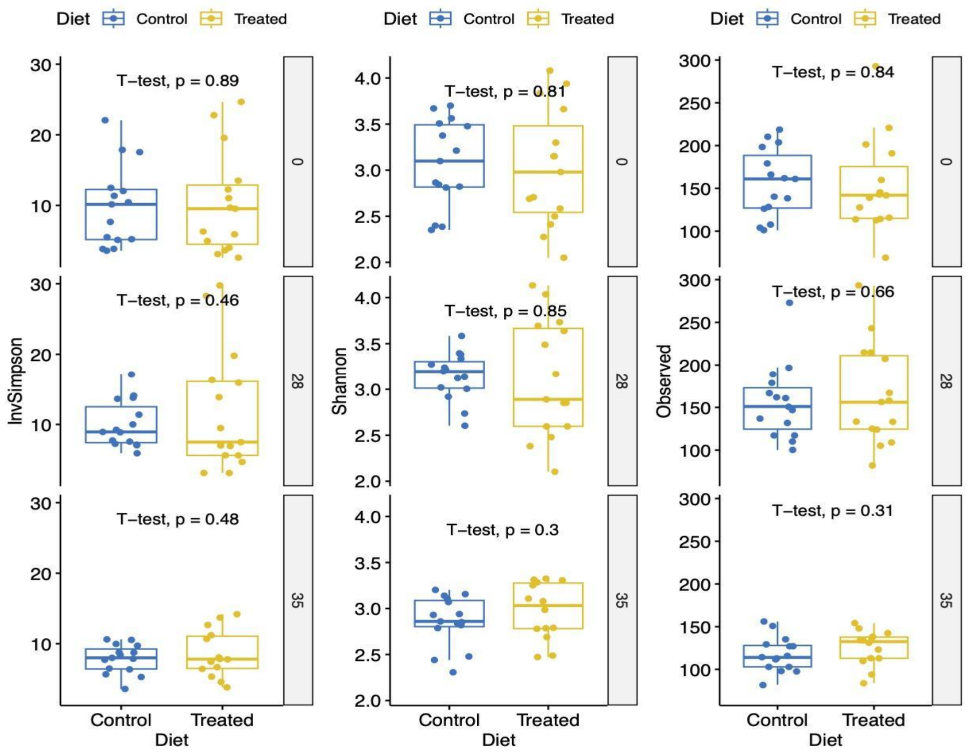

Supplementary Figure S2: Alpha diversity analysis of treated vs control groups at T0, T28 and T35.

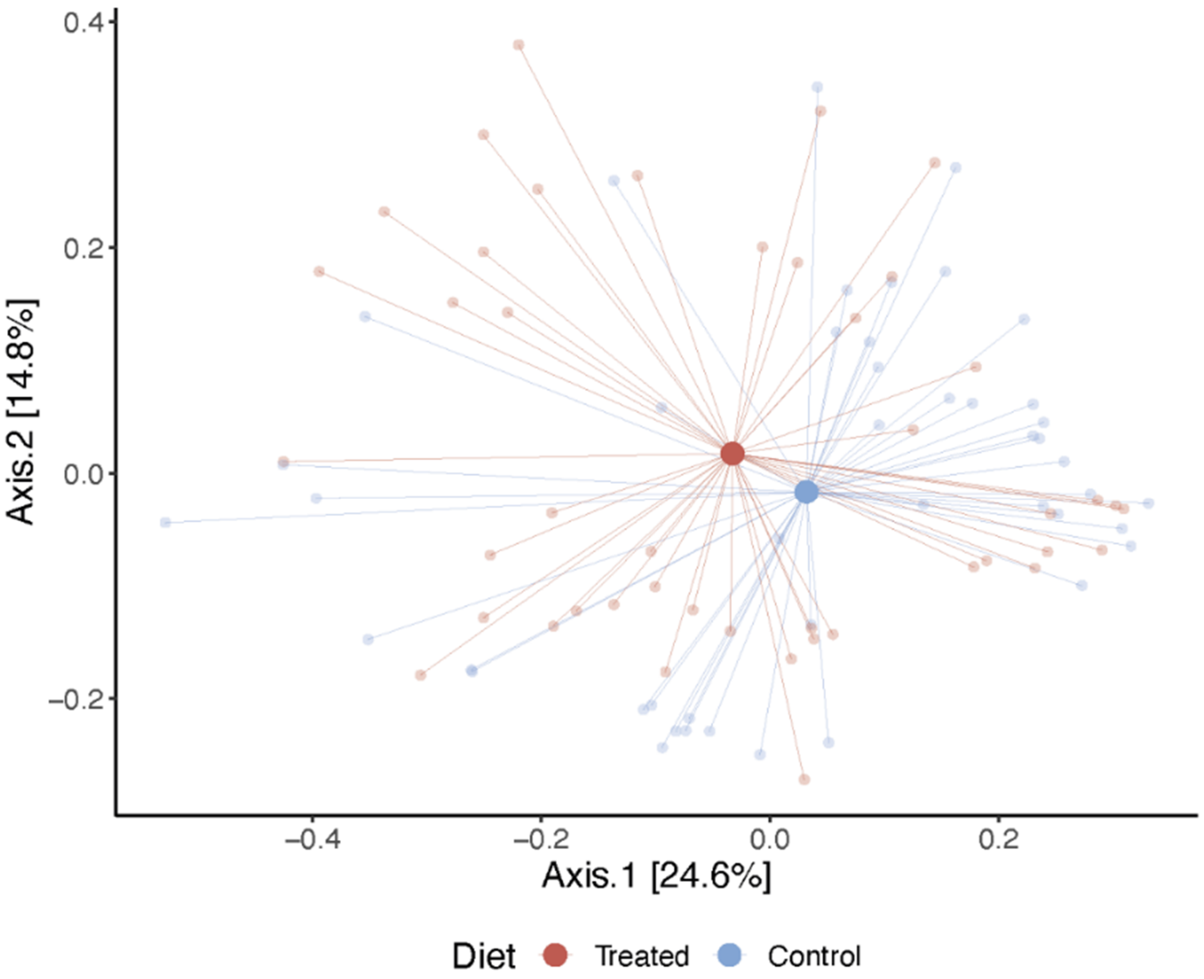

Supplementary Figure S3: A principal coordinate analysis using Bray-Curtis dissimilarity matrix for β diversity colored by treated and control groups.
